# A Clinico-Pathologic Study of 82 Intraoral Minor Salivary Gland Tumors

**DOI:** 10.5812/kowsar.20741804.2244

**Published:** 2011-09-15

**Authors:** Z Jaafari-Ashkavandi, M J Ashraf, N Afandak

**Affiliations:** 1 Department of Oral Pathology, School of Dentistry, Shiraz University of medical Sciences, Shiraz, Iran; 2 Department of Pathology, Khalili Hospital, Shiraz, Iran; 3 Dentistry School, Shiraz University of medical Sciences, Shiraz, Iran

**Keywords:** Salivary glands, Minor, Tumor, Iran

## Abstract

**Background:**

Intraoral minor salivary glands tumors (MSGT) are uncommon, with geographic variations in frequency and distribution. This study analyzed the clinic-pathologic features of these tumors in Shiraz, southern Iran.

**Methods:**

All of cases histopathologically recorded as epithelial MSGT in Pathology Department of Khalili Hospital from 2002 to 2009 were evaluated with regard to patient's age and gender, tumor location and type, retrospectively.

**Results:**

Out of 82 cases of MSGT, 53.7% were benign and 46.3% were malignant. Pleomorphic adenoma and adenoid cystic carcinoma were the most common benign and malignant tumors, respectively. The palate was the most common site of involvement (64.6%). Male to female ratio was 1:1.27. The mean age of the patients was 35.0±17.2 years for benign and 48.8±18.2 years for malignant tumors.

**Conclusion:**

MSGTs in the present study represent many characteristics of other studies. However, our patients who were affected by benign tumors were young.

## Introduction

Minor salivary Minor salivary glands are functional structures located in the submucosa of the upper aerodigestive tract.[1] The importance of the lesions lies in the fact that lesions are more likely to be malignant.[2] Studies from other countries report that 13.9-51.4% of all salivary gland tumors (SGTs) arise from an intraoral area and 34.7-67.1% of them are benign.[3][4][5][6] It was shown that incidence of SGTs is influenced by geographic and racial factors.[7][8] Up to now, a large series of MSGTs were reported in Iran. The aim of this study was to determine clinic-pathological features of 82 cases of benign and malignant intraoral MSGT in this region.

## Materials and Methods

All of 82 histopathological approved cases recorded as epithelial SGT in the oral mucosa and jaws location in Pathology Department of Khalili Hospital, ENT center affiliated to Shiraz University of Medical Sciences in Southern Iran, from 2005 to 2009 were enrolled. Recurrent, metastatic and non-epithelial neoplasms were excluded. The cases were evaluated with regard to patient's age and gender, tumor location and histopathologic type according to the 2005 WHO classification, through a retrospectively review of the patients’ medical files. They were analyzed using SPSS software (version 11.5, Chicago, IL, USA).

## Results

**Fig. 1:**
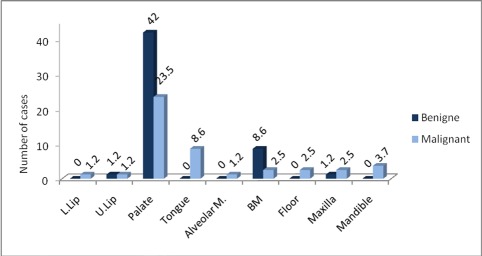
Location of benign and malignant tumors. L: lower, U: upper, M: mucosa, BM: Buccal mucosa

Among 356 SGTs, 82 cases (23%) were recorded as epithelial SGT in the oral mucosa and jaws. Forty four (53.7%) were benign and 38 cases (46.3%) were malignant (Table 1). The patients were between 13 to 80 years, with a mean of 41.4±18.9. The palate was the most common site of involvement in both benign and malignant types, corresponding to 64.6% of all cases (Figure 1). Five cases of tumors (7.4%), including one case of pleomorphic adenoma (PA), one case of polymorphous low-grade adenocarcinoma (PLGA) and 3 cases of mucoepidermoid carcinoma (MEC) were centrally located in the maxilla and mandible. Overall male to female (M/F) ratio was 1:1.27. Table 1 shows the type, age and gender distribution of tumors.

**Table 1:** Type, age and gender distribution of minor salivary glands^a^

**Tumor type**	**Frequency (%)**	**M:F**	**Mean age ± SD**	**Age range**
Benign tumors	44 (53.7)	22:22	35 ± 17.25	13–72
PA	36 (43.9)	16:20	34.5 ± 2.7	13–72
Myoepithelioma	7 (8.5)	6:1	39 ± 8.5	19–71
Monomorphic adenoma	1 (1.2)	0:1	23	23
Malignant tumors	38 (46.3)	36:46	48.8 ± 18.1	13–80
MEC	10 (12.2)	3:7	43.7 ± 5.2	15–63
ACC	1 (1.2)	0:1	60	60
AdCC	23 (28.0)	10:13	50.8 ± 4	13–80
ca.ex.PA	1 (1.2)	0:1	67	67
PLGA	3 (3.7)	1:2	41 ± 7.6	26–51
Total	82 (100.0)	58:68	41.4 ± 18.9	13–80

^a^ PA: pleomorphic adenoma, MEC: mucoepidermoid carcinoma, ACC: acinic cell carcinoma, AdCC: adenoid cystic carcinoma, ca.ex.PA: carcinoma ex pleomorphic adenoma, PLGA: polymorphous low-grade carcinoma

## Discussion

To the present time, little data is available on the clinic-pathologic presentation of MSGTs in Iran. We studied these cases in an ENT center during a 5 years period. Among 9700 cases, MSGTs were uncommon, representing 0.84% of all the biopsy specimens. Intraoral MSGTs accounted for 0.28-1.9% of the specimens, as reported by other authors in USA, Japan, Venezuela and Thailand.[4][9][10][11]

**Fig. 2:**
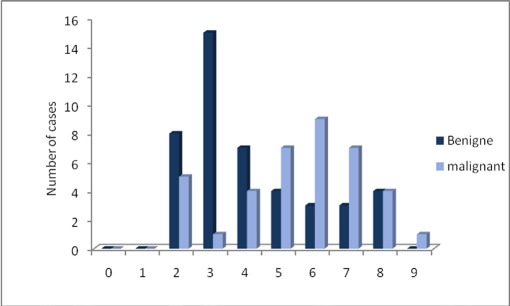
Incidence of benign and malignant tumors in each decade

In our series, malignant neoplasms constituted 46.3% of the cases which is in agreement with the results of the most previous studies.[5][9][12] However, some have shown a predominance of malignant tumors.[6][9][13][14][15] Most of these researches performed in special situations such as cancer registry and cancer treatment centers. In the current study, PA was the most common tumor, representing 43.9% of all and 81.8% of the benign tumors, which is comparable with other series that reported the incidence of this entity from 15.814 to 78 %16of cases (Figure 2).[2][3][4][5][6][9][10][11][14][16]

Among malignant tumors, Ad CC was the most common. Figure 2 is similar to some reports,14,15,17 but the authors in USA, Brazil, Venezuela, Australia, China and Ansari on 18 cases of MSGTs in central Iran, reported that MEC was the most frequent.[6][10][16][18][19][20][21]

According to these data, there is a geographic variation in relative frequency of Ad CC versus MEC. However, these two neoplasms are the first and second malignant tumors in the most series.

The palate was the most common site of involvement in our findings (64.6%), followed by buccal mucosa. The palate accounted for 34.6 [21]- 82.9%11 of cases in previous studies. This location was the most commonly affected site for both benign and malignant neoplasms, but benign tumors were predominant. This finding is in agreement with other studies.[3][4][11][20] Our results showed that the tongue (8.5%) and jaws (7.3%) were the next sites. Similar to our findings, Dhanuthai et al., and Jabber et al. reported 6.5% and 5.2% of MSGTs in the jaws.[11][21]

The peak prevalence of benign tumors occurred in the third decade of life and that of malignant types were in the 6th and 7th. Really, the mean age of malignant tumors was 13.8 years later than benign ones. This difference was higher in our population than the previous studies, reporting a range from 2.9 years in Thais to 13.3 years in African population.[11][22] This finding is due to the fact that in our series, patients with benign tumors were younger (mean age: 35) while others have stated that the mean age of the affected patients with these neoplasms was from 40 in Chinese to 49 in Japanese.[3][4]

Overall, the incidence of MSGTs was higher in females (M/F ratio was 1:1.27). This ratio was reported from 1:1.02[15] to 1:2.[10] It has been suggested that gender distribution in SGTs is related to ethnic variations.[4][22]

In conclusion, in the present population, PA was the most common neoplasm, the palate was the most commonly affected site, and female tendency of MSGTs was seen. Ad.CC was the most common malignancy as some other Asian nations. However, our patients affected by benign tumors were younger. Our findings could help us to perform better diagnosis and early treatment for this group of patients.
